# Individual Beef Cattle Identification Using Muzzle Images and Deep Learning Techniques

**DOI:** 10.3390/ani12111453

**Published:** 2022-06-04

**Authors:** Guoming Li, Galen E. Erickson, Yijie Xiong

**Affiliations:** 1 Department of Agricultural and Biosystems Engineering, Iowa State University, Ames, IA 50011, USA; gmli@iastate.edu; 2 Department of Animal Science, University of Nebraska-Lincoln, Lincoln, NE 68583, USA; gerickson4@unl.edu; 3 Department of Biological Systems Engineering, University of Nebraska-Lincoln, Lincoln, NE 68583, USA

**Keywords:** animal biometrics, cognitive science, computer vision, machine learning, precision livestock management, pattern recognition

## Abstract

**Simple Summary:**

The ability to identify individual animals has gained great interest in beef feedlots to allow for animal tracking and all applications for precision management of individuals. This study assessed the feasibility and performance of a total of 59 deep learning models in identifying individual cattle with muzzle images. The best identification accuracy was 98.7%, and the fastest processing speed was 28.3 ms/image. A dataset containing 268 US feedlot cattle and 4923 muzzle images was published along with this article. This study demonstrates the great potential of using deep learning techniques to identify individual cattle using muzzle images and to support precision beef cattle management.

**Abstract:**

Individual feedlot beef cattle identification represents a critical component in cattle traceability in the supply food chain. It also provides insights into tracking disease trajectories, ascertaining ownership, and managing cattle production and distribution. Animal biometric solutions, e.g., identifying cattle muzzle patterns (unique features comparable to human fingerprints), may offer noninvasive and unique methods for cattle identification and tracking, but need validation with advancement in machine learning modeling. The objectives of this research were to (1) collect and publish a high-quality dataset for beef cattle muzzle images, and (2) evaluate and benchmark the performance of recognizing individual beef cattle with a variety of deep learning models. A total of 4923 muzzle images for 268 US feedlot finishing cattle (>12 images per animal on average) were taken with a mirrorless digital camera and processed to form the dataset. A total of 59 deep learning image classification models were comparatively evaluated for identifying individual cattle. The best accuracy for identifying the 268 cattle was 98.7%, and the fastest processing speed was 28.3 ms/image. Weighted cross-entropy loss function and data augmentation can increase the identification accuracy of individual cattle with fewer muzzle images for model development. In conclusion, this study demonstrates the great potential of deep learning applications for individual cattle identification and is favorable for precision livestock management. Scholars are encouraged to utilize the published dataset to develop better models tailored for the beef cattle industry.

## 1. Introduction

Beef products are among the most consumed animal protein, and the beef cattle industry is critical for many rural communities. Numerous challenges face the beef industry including needs for improved nutrition and production efficiency to feed a growing human population [1,2]. The US is the largest beef producer, the second-largest importer, and the third-largest exporter by volume, and it has the largest total consumption globally [3]. During 2021, the total US cattle inventory (including all cattle and calf operations) was 93.6 million heads with an annual cash receipt of 66.0 billion USD, accounting for 16.9% of the 391 billion USD in total cash receipts from all agricultural commodities [3,4]. As a proportion of total beef produced, feedlots contribute approximately 77% of the cattle marketed in the US [5,6,7], with the remainder as beef slaughtered from culled cows/bulls. US feedlots with a capacity of more than 1000 heads market 80–85% of all fed cattle [3]. Maintaining and inspecting the intensive production systems daily to the individual animal level is a continual challenge for feedlot producers [8,9]. The value of individual cattle can vary but is significant, with prices of feeder cattle at 700–1000 USD per 227 kg calf [10] and 1500–2000 USD for mature cattle at market. Identifying individual cattle accurately and efficiently will increase the producer’s viability in establishing ownership, developing methods for individual tracking for health management and disease tracking, and providing individual traceability throughout the production chain [11,12].

Cattle identification can be performed using contact and noncontact methods [11], with branding commonly used to establish ownership. Common contact methods include ear notching, ear marking, ear tagging, and branding. While these methods display clear identification of individual cattle, human efforts are always required to recognize and locate the cattle, which is laborious and time-consuming. Another commonly used method, radiofrequency identification (RFID) systems, may overcome the drawback. To use an RFID system, an RFID transponder needs to be attached to each cattle and is read automatically and continuously by a reader. In some countries, RFID tags are extensively and mandatorily used in modern beef production systems (e.g., Australia). However, like other contact methods, in practice, such systems may cause cattle stress and are prone to damage once attached to the animals. The sensors (e.g., tags, transponders) with animal identification can fade, be damaged, and be lost due to cattle interference, movement, and environmental exposure [13]. Contact methods may also lead to short- and long-term complications in the integrity of cattle ears or other anatomical body parts [12].

**Figure 1:**
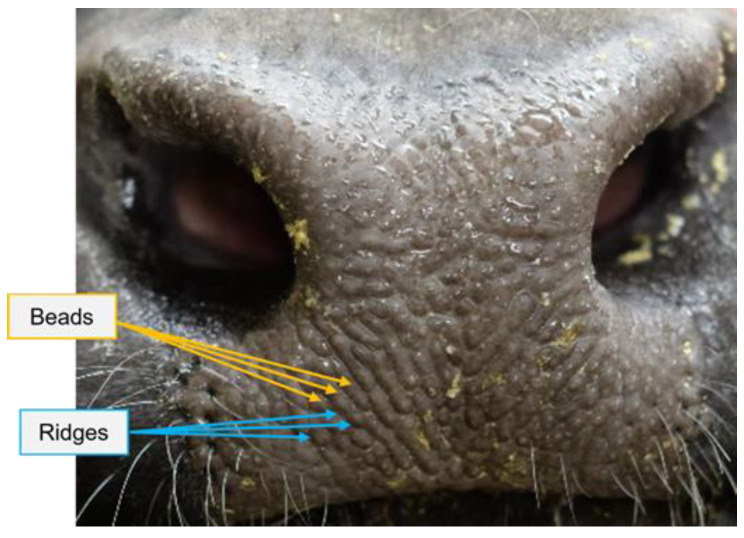
The illustration and terminologies of a beef cattle muzzle pattern.

Alternatively, contactless identification methods may eliminate human disturbance to the animals and use unique animal biometric features. Means of identifying livestock biometric markers include DNA pairing, autoimmune antibody matching, iris scanning, retinal imaging, coat pattern recognition, muzzle identification, and facial recognition [14]. These biometric markers or modalities have phenome and genome characteristics that are unique to the individual animal, tamperproof over time, invariant to transformation, and welfare-friendly to animals [15]. Among them, muzzle identification is a relatively low-cost and simple method and has recently received increasing research interest. Muzzle pattern is a cattle dermatoglyphic trait equivalent to human fingerprints. The little round-, oval-, or irregular-shaped protuberances on the nose area are defined as beads, while the elongated grooves and valleys arranged in a particular manner are defined as ridges (Figure 1). These uneven and distinct features make the muzzle identification for individual cattle possible.

A reference summary of previous cattle muzzle identification studies was thoroughly conducted to investigate the method development progress and determine the current research gaps (Table 1). Petersen [16] was the first to explore the muzzle pattern recognition for dairy cattle. During the early stages [16,17,18], investigators manually observed imprinted muzzle patterns and explored the muzzle uniqueness of healthy and ill cattle. These studies contributed significantly to examining the possibility of muzzle recognition; however, manual observation was laborious and not suitable for large-scale application. Then, conventional digital image processing algorithms (e.g., scale-invariant feature transform and box-counting fractal dimension models) were used to identify individual cattle automatically [15,19]. These methods typically matched features, including color, texture, shape, and edge, among different muzzle images and achieved high identification accuracy (e.g., 98.3% [20] and 100% [21]) with small image sets and controlled conditions. However, the method performance may be challenged by inconsistent illumination and background, variable muzzle shapes and sizes, similar appearances of the same animal at different times, missing parts or occlusions on a muzzle, and low resolution [22]. Machine learning classification models (e.g., support vector machine, K-nearest neighbor, and decision tree) were embedded with image processing-based feature extractors (e.g., Weber local descriptor) to further boost the performance of muzzle identification [23,24,25]. Despite promising results with an over 95% accuracy for beef cattle muzzle classification, the approaches require sophisticated hand-crafted features and may be difficult to develop and optimize for researchers from non-computer science backgrounds.

Deep learning is a data-driven method and has been researched for computer vision applications in animal production [22]. Deep learning models can capture spatial and temporal dependencies of images/videos through the use of shared-weight filters and can be trained end-to-end without strenuous hand-crafted design of feature extractors [26], empowering the models to adaptively discover the underlying class-specific patterns and the most discriminative features automatically. Kumar et al. [27], Bello et al. [28], and Shojaeipour et al. [29] tried deep learning models (e.g., convolutional neural network, deep belief neural network, You Only Look Once, and residual network) in large sets (over 2900 images in total) of dairy and beef cattle muzzle images and obtained great accuracies of over 98.9%. The US beef cattle industry is quite unique from the dairy sector, in terms of both animal genetics and housing environment [3], which may result in different bioinformatic markers between dairy and beef cattle that influence model classification performance. Shojaeipour et al. [29] investigated the muzzle pattern of beef cattle, but the cattle were constrained in a crush restraint (i.e., hydraulic squeeze chute) with their heads placed in head scoops for data collection, which may cause extra distress to the cattle. Moreover, except for the study of Shojaeipour et al. [29], the muzzle image datasets were not publicly available in most studies, limiting the progress of developing models tailored for beef cattle applications.

Model processing speed was only reported in a limited number of publications [15,23,30] but not reported in the three recent deep learning studies mentioned above (Table 1). Processing speed is a critical metric to estimate the overall identification duration in a farm when classification models are incorporated into computer platforms or robots for use. During conventional data collection [16], cattle were constrained with ropes or other tools, snot and grass on the nose was wiped clean with tissues, thin ink was smeared on the nose area, the paper was rolled upward or downward to obtain the printed muzzle pattern, and the imprinted muzzle was scanned or photographed to digitalize the muzzle pattern for further data analysis. Such procedures may acquire clear muzzle patterns but are also complicated and inefficient to apply in modern feedlots. Grayscale images were applied in some studies but only provided one-channel information, whereas RGB (red, green, and blue) images contain richer information for processing and were applied more frequently, as indicated in Table 1.

**Table 1:** Reference summary from previous cattle muzzle identification studies.

Cattle Type	Image Size (Pixels)	Image Type	Restrained	Cattle Counts	Images per Cattle	Total Images	Identification Method	Accuracy (%)	Processing Time (ms/Image)	Reference
Dairy	−	Printed	Y	−	−	6	Manual	−	−	[16]
−	−	Printed	Y	−	−	200	Manual	−	−	[17]
−	−	Printed	Y	65	−	−	Manual	−	−	[18]
Beef	256 × 256	Grayscale	Y	−	−	43	DIP	46.5	−	[31]
Beef	320 × 240	Printed	Y	29	10	290	ML	98.9	−	[12]
Beef	200 × 200	Grayscale	−	8	10	80	DIP	90.0	−	[32]
−	−	Grayscale	−	15	7	105	DIP	93.3	37–879	[15]
Beef	−	Printed	Y	20	8	160	DIP	98.3	−	[20]
−	−	Grayscale	−	53	20	1060	DIP	−	−	[19]
Beef	300 × 400	Grayscale	−	31	7	217	ML	99.5	−	[33]
−	−	RGB	−	28	20	560	ML	100.0	−	[25]
−	−	RGB	−	52	20	1040	ML	96.0	−	[24]
Beef	−	RGB	N	14	5	70	DIP	100.0	−	[21]
Beef	300 × 400	Grayscale	−	31	7	217	ML	99.5	−	[34]
Beef	300 × 400	Grayscale	−	31	7	217	ML	99.5	48–1362	[23]
Beef	−	RGB	−	52	6	312	ML	96.4	−	[35]
Dairy	400 × 400	RGB	−	500	10	5000	DIP	93.9	−	[36]
Dairy	200 × 200	RGB	−	500	10	5000	ML	94.9	−	[37]
Dairy	200 × 200	RGB	−	500	10	5000	DL	98.9	−	[27]
Dairy	200 × 200	RGB	−	500	10	5000	ML	93.9	−	[38]
Dairy	−	RGB	N	15	7	105	ML	93.0	368–1193	[30]
Beef	−	RGB	Y	60	5–10	460	DIP	98.1	−	[39]
Beef	−	RGB	−	45	20	900	ML	96.5	−	[40]
Beef	−	RGB	Y	431	−	1600	ML	95.0	−	[41]
Dairy	200 × 200	RGB	−	400	10	4000	DL	98.9	−	[28]
Beef	1024 × 1024	RGB	Y	300	−	2900	DL	99.1	−	[29]
Dairy	64 × 64	RGB	−	186	5	930	ML	83.4	−	[13]

Note: ‘−’ indicates that information was not provided. DIP, digital image processing; ML, machine learning; DL, deep learning. Cattle species include beef cattle and dairy cattle. Image type is categorized as printed (samples are obtained from a direct compress with cattle noses and then scanned or photographed to form electronic images), grayscale with one-channel data captured directly from cameras, and RGB with three-channel (red, green, and blue) data. ‘Y’ indicates that the animal was restrained during data collection, while ‘N’ indicates that it was not.

The objectives of this study were to (1) collect high-resolution RGB muzzle images of feedlot cattle without any restraint or contact with the animals to develop a high-quality dataset to train deep learning models for individual cattle identification, and (2) benchmark classification performance and processing speed of muzzle identification optimized with various deep learning techniques.

## 2. Materials and Methods

### 2.1. Image Collection and Dataset Curation

This research was conducted at the University of Nebraska-Lincoln (UNL) Eastern Nebraska Research Extension and Education Center (ENREEC) research farm located near Mead, NE, USA. All animals were cared for under approval of the UNL Institution of Animal Care and Use Committee protocol 1785 (approved 4 December 2019), and no direct contact with animals was made throughout the course of data collection.

The RGB images of beef cattle were collected using a mirrorless digital camera (X-T4, FUJIFILM, Tokyo, Japan) and a 70–300 mm F4-5.6 focal lens (XF70-300 mm F4-5.6 R LM OIS WR, FUJINON, Tokyo, Japan), from 11 March to 31 July 2021. All images were collected from various distances outside the pens, while cattle were free to express their natural behaviors. A total of 4531 raw images from 400 US mixed-breed finishing cattle (*Angus*, *Angus* × *Hereford*, and *Continental* × *British* cross) were collected, of which the muzzle areas were the focus of the images. The ear tag information of each animal was recorded for verifying individual beef cattle. Because all images were taken under natural outdoor feedlot conditions, these images were presented with different angles of view and lighting conditions.

**Figure 2:**
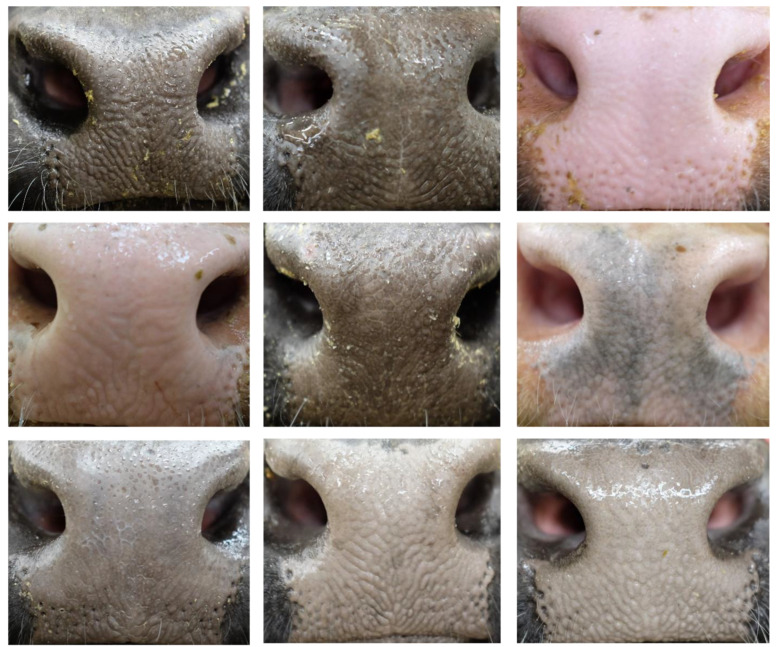
Sample muzzle images of nine individual mixed-breed beef cattle.

Raw images contained unnecessary anatomical parts (e.g., face, eye, and body), particularly for classification purposes. To reduce classification interference and highlight muzzle visual features, the cattle face area was rotated so that the muzzle area aligned horizontally, after which the muzzle area was manually cropped. Extremely blurry, incomplete, or feed-covered muzzle images were removed to maintain dataset quality. Small sets of images per animal (≤3) were also discarded to obtain sufficient data for model training, validation, and testing. At the end, a total of 4923 muzzle images (multiple muzzles could be cropped from a single raw image) from 268 beef cattle were selected to form the dataset. Nine sample images from nine cattle are presented in Figure 2. Although colors and textures could be similar among individuals, the patterns of beads, grooves, and ridges were visually different, which were favorable to individual identification. The cropped muzzle images are published in an open-access science community [42].

**Figure 3:**
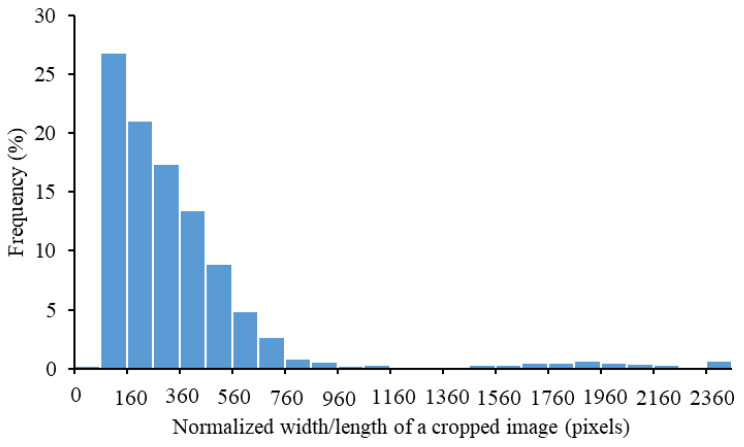
Frequency distribution of normalized width/length of a cropped image.

A frequency distribution of the normalized width/length of cropped images is depicted in Figure 3. Inconsistent image sizes may lead to biased detection performance, while high-resolution (large-size) images can downgrade the processing efficiency [43]. Therefore, the cropped images were resized to the same dimensions before being supplied into classification models. The dimensions should be determined on the basis of the following criteria: (1) with similar dimensions to those reported in the previous studies (Table 1); (2) with greater frequency of normalized width/length of the cropped muzzle images in the dataset, as indicated in Figure 3; (3) compliance with the input size requirement of most deep learning image classification models. In the end, dimensions of 300 × 300 pixels were selected in this study to normalize the cropped images.

### 2.2. Deep Learning Image Classification Models

A total of 59 deep learning image classification models were comparatively evaluated to determine the optimal models for identifying individual beef cattle with the cropped and resized muzzle images (Table 2). These models came from 14 major image classification model families, which were AlexNet [44], DenseNet [45], DPN [46], EfficientNet [47], Inception [48,49,50,51], MnasNet [52], MobileNet [53,54], RegNet [55], ResNet [56], ResNeXt [57], ShuffleNet [58], SqueezeNet [59], VGG [60], and Wide ResNet [61]. Total parameters and model sizes of these models ranged from 1.2 to 145 million and from 2.5 to 543.7 MB, respectively. These models were from the PyTorch (a popular deep learning platform accelerating big data analytics)-based libraries, TORCHVISION (https://pytorch.org/vision/stable/models.html, accessed on 10 March 2022), and PRETRAINEDMODELS (https://pypi.org/project/pretrainedmodels, accessed on 10 March 2022). Other models, if available or newer, were either incompatible with the PyTorch operation environment or unsuitable for the resized muzzle images (300 × 300 pixels). All models were expected to be evaluated with the same operation environment to reduce environment interference; therefore, these models were not considered in this study.

### 2.3. General Model Evaluation and Development Strategies

Transfer learning was deployed during training, with which models were pre-trained with a large dataset, ImageNet [62], whereas only the fully connected layers of the models were fine-tuned with the current dataset for custom classification. This strategy improves training efficiency without compromising inference performance. The cattle muzzle dataset was randomly partitioned and reshuffled into three subsets: 65% for training, 15% for validation, and 20% for testing. Image pixel intensities per color channel were normalized to the range of [0,1] for enhanced image recognition performance [63]. Each model was trained with five replications assigned the same random seeds, and the mean accuracy on the testing dataset was computed to evaluate model performance and reduce the random effects resulting from data reshuffling. All models were trained for 50 epochs (in which training typically converged for muzzle data), using a stochastic gradient descent optimizer and momentum of 0.9. The learning rate was initially set to 0.001 and dynamically decayed by a factor of 0.1 every seven epochs for stabilizing the model training. Models were trained and validated in a cloud-based service, Google Colab Pro, allocated with a Tesla P100-PCIE-16GB GPU, 12.69 GB of RAM, and a disk space of 166.83 GB. The working space and ethernet speed of cloud services can vary, resulting in inconsistent processing speeds among different models. Therefore, a local machine with an Intel^®^ Core™ i7-8700K CPU @ 3.70 GHz processor, 16.0 GB of RAM, and Windows 10^®^ 64 bit operation system was also used for model testing. Utilization of multiple machines allowed accelerating training speed with the cloud-allocated GPU and examining standard model performance without the GPU for mobile applications.

The cross-entropy (CE) loss function was used to evaluate the training and validating model performance during the training process (Equation (1)).

(1) $$CE_{loss}=-\overset{C}{\underset{i=1}{\sum }}wt_{i}\log\left(p_{i}\right),$$

where $p_{i}\in \;{\mathbb{R}}^{268}$ (${\mathbb{R}}^{268}$ indicates a 268-dimensional vector) is the vector of a Softmax output layer and indicates the probability of predicting the 268 individual cattle, C is the number of cattle (=268), w (=1) indicates that equal weights were assigned to all cattle, and $t_{i}$ denotes the true probability for the *i*-th cattle, is defined as follows:

(2) $$t_{i}=\{\begin{array}{c} \begin{array}{cc} 1, & if\;i=true \end{array}\\ \begin{array}{cc} 0, & otherwise \end{array} \end{array}.$$

Accuracy was calculated for each model during each epoch of training using the validation dataset and after training using the testing dataset to determine model performance for overall classification. It was also calculated for each class to determine individual identification performance. Processing speed was computed using the reported time in Python divided by total number of processed images. Higher values suggest better model accuracy but lower processing speed. We proposed a comprehensive index (CI, Equation (3)) to balance the two opposite evaluation metrics to determine comprehensive performance for each model. The accuracy and processing speed computed from the testing dataset was firstly ranked, where high accuracy values and low processing speeds had high orders. Because accuracy was considered more important than processing speed in this study, 80% of the ranked results were weighed for accuracy while 20% were weighed for the processing speed. The proportion can be changed on the basis of the specific metric importance determined by developers. Overall, a lower CI indicates that the model provides better comprehensive performance.

(3) $$CI=80\%\times Order{\;}_{accuracy}+20\%\times Order_{proessing\;speed},$$

where the variable $Order_{i}$ represents integers ranging from 1 to 59, and the subscripts refer to the appropriate metric of interest.

Pearson’s correlation analysis was conducted to understand the correlation effects among model total parameters, model size, accuracy, and processing speed. Larger absolute values of the Pearson correlation coefficient (*R*) indicate a higher correlation between parameters.

### 2.4. Optimization for Class Imbalance

Class imbalance was observed in the cropped muzzle image dataset with a range of images per cattle from four to 70 (Table 3). Because fewer images were fed to the dataset, a minority class (cattle with fewer images) may be prone to misidentification. Two commonly used deep learning strategies, weighted cross-entropy (WCE) loss function [64] and data augmentation [22], were adopted to mitigate this issue during model training. Both optimization strategies were evaluated by 20 models, which were determined using the optimal accuracy, processing speed, and CI among the 59 models. Accuracy was the primary metric to optimize class imbalance.

The WCE loss function assigned heavier weights for cattle with fewer cropped muzzle images, as defined in Equation (4).

(4) $$WCE_{\text{loss}}=-\overset{C}{\underset{i=1}{\sum }}w_{i}t_{i}\log\left(p_{i}\right)$$

where $w_{i}$ is the individualized weight assigned to the *i*-th cattle, which can be calculated as follows:

(5) $$w_{i}=\frac{N_{max}}{N_{i}},$$

where $N_{i}$ denotes the image number for the *i*-th cattle, and $N_{max}$ is the maximum image counts per head (=70 in this case). The assigned weight in the WCE loss function for each cattle is provided in Table A1 in Appendix A.

Data augmentation is a technique to create synthesized images and increase limited datasets for training deep learning models. The augmentation was implemented as a preprocessing step in an end-to-end training or interference process. Four augmentation strategies were adopted on the basis of raw image limitations, namely, horizontal flipping, brightness modification, randomized rotation, and blurring. The horizontal flipping was to mimic events when cattle were photographed in different locations due to their natural behaviors. The brightness modification was to mimic varying outdoor lighting conditions, and the brightness factor was set from 0.2 to 0.5 (minimum = 0, maximum = 1). Rotation was randomized from −15° to 15°, simulating natural cattle head movements. Blurring was applied to simulate the cases of overexposure and motion blur, and a Gaussian function with kernel sizes of 1–5 was used to create blurred muzzle images.

## 3. Results and Discussion

### 3.1. Examples of Validation Performance

**Figure 4:**
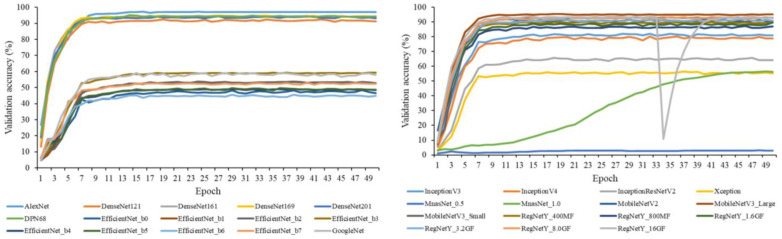

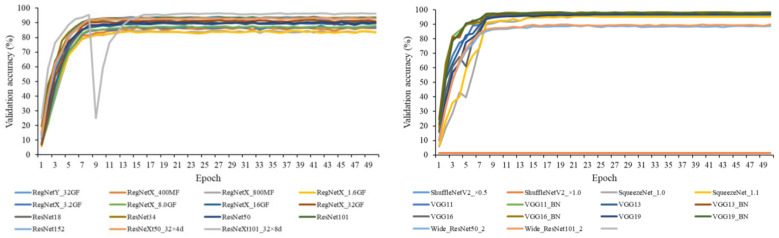
Validation accuracy of the 59 deep learning image classification models. Descriptions of the models are provided in Table 2.

Figure 4 provides representative results of the validation accuracy of the 59 deep learning image classification models assessed. Most models converged before the 50th epoch, and various models achieved the best validation accuracy at an epoch between 13 to 49. Each model took 20 to 302 min to finish the training of 50 epochs, using the cloud service. MnasNet_0.5, ShuffleNetV2_×0.5, and ShuffleNetV2_×1.0 consistently showed a low validation accuracy (<5%) across all training epochs. Valley points were observed for validation accuracy curves of RegNetY_16GF and RegNetX_800MF, probably because of data randomness after data reshuffling. In sum, the 50 epochs were reasonable configurations for model training, and validation accuracy was observed for all model training, including the training of 20 selected models for optimizing the accuracy of individual identification.

### 3.2. Testing Performance of the Selected Deep Learning Image Classification Models

Table 3 shows the testing accuracies and processing speeds of the 59 deep learning image classification models. Accuracy ranged from 1.2% to 98.4% with ShuffleNetV2_×0.5 being the worst and VGG16_BN being the best. Each model took 32.3 to 678.2 ms to process a muzzle image, with the ShuffleNetV2_×0.5 being the fastest model and EfficientNet_b7 being the slowest model. Twenty models were selected and organized on the basis of the CI ranking, namely, VGG11_BN, AlexNet, VGG16_BN, VGG13, SqueezeNet_1.1, VGG11, VGG13_BN, MobileNetV3_Large, VGG19_BN, VGG16, SqueezeNet_1.0, VGG19, MobileNetV3_Small, ResNeXt101_32×8 d, ResNet34, DenseNet169, DPN68, DenseNet161, DenseNet201, and RegNetY_32GF. The 20 models were further used to evaluate the optimization of class imbalance.

The processing speed was computed by Google Colab (with GPU) for all 59 models, and the average ± standard deviation was 60.5 ± 93.4 ms/image, much lower than the speed 197.8 ± 145.1 ms/image computed by the local computer with CPU only (Table 3), presumably due to the cloud-based service. For example, the processing speed using the cloud was 103.6 ms/image for DenseNet121 but 20.9 ms/image for DenseNet161, 182.4 ms/image for EfficientNet_b3 but 12.4 ms/image for EfficientNet_b4, 379.0 ms/image for MnasNet_0.5 but 10.0 ms/image for MnasNet_1.0, and 186.2 ms/image for VGG13 but 18.8 ms/image for VGG16. The internet speed inconsistency may have led to the abnormal data trends where simpler architectures in the same model family processed images more slowly. Therefore, although the current processing speed provided with CPU only was not optimal, it was at least reliable for building the benchmark performance for some mobile computing machines without GPU.

Table 4 presents a Pearson correlation coefficient (*R*) matrix to better understand the relationships among model performance parameters. Accuracy had a low and positive correlation with model parameters (total parameter and size), while processing speed was moderately and positively correlated with model parameters. This result matched with our original hypothesis of correlation direction (a complicated model with more model parameters should have greater accuracy but longer processing time), although we expected greater correlation magnitudes. More factors may also affect model performance, such as connection schemes and network depth. For example, both ShuffleNet [58] and SqueezeNet [59] were lightweight models with 1.2–2.3 million total parameters, a 2.5–6.0 MB model size, and a fast processing speed of 32.3–62.1 ms/image. However, SqueezeNet achieved much better accuracies (95.0–95.9%) than that of ShuffleNet (1.2–1.3%). SqueezeNet introduced the Fire module (a squeezed convolution filters) to build CNN architecture and achieved AlexNet-level accuracy with fewer total parameters and smaller model sizes [59]. ShuffleNet used a direct measure of processing speed rather than an indirect measure of FLOPs to efficiently design and optimize CNN architecture, although the method was not beneficial in improving accuracy (the top-1 error rate was up to 39.7% [58]).

Interestingly, a few earlier models, such as AlexNet [44] and VGG [60], outperformed some newer models (e.g., EfficientNet [47], MnasNet [52], and RegNet [55]). One plausible explanation is that the connection scheme greatly impacted the model performance for this muzzle image dataset. AlexNet and VGG were operated in a feed-forward manner and could improve model performance by increasing architecture depth, while other models increased architecture width, introduced shortcut connection, and scaled up architecture width and depth. Our results indicate that a simple and feed-forward network architecture is sufficient in identifying individual beef cattle using muzzle images.

### 3.3. Optimization Peformance for Class Imbalance

The highest accuracy of the 20 selected models increased by 0.1% with the weighted cross-entropy loss function and 0.3% with data augmentation, compared with that of the development without any class imbalance optimization (98.4%, Table 5). The average accuracy increased by 0.6% with weighted cross-entropy but decreased by 0.2% with data augmentation, compared with the development without any class imbalance optimization (96.1%, Table 5). It turned out that the accuracy was not consistently improved for every model by the class imbalance optimization (e.g., AlexNet, MobileNetV3_Small, SqueezeNet_1.0, SqueezeNet_1.1, and VGG13). Therefore, only the models that performed best in both strategies, VGG16_BN (with cross-entropy loss function and data augmentation) and VGG19_BN (with weighted cross-entropy loss function), were selected to evaluate the accuracy of individual cattle identification.

There was no significant difference in processing speed between the developments conducted with cross-entropy and with weighted cross-entropy. However, the processing speeds of the 20 models with data augmentation were faster compared to those without any class imbalance optimization. The processing time was the sum of model loading time and total image processing time divided by the total number of processed images (Equation (6)). The model loading time was the duration of loading models to the CPU machine and ranged from 95.7 to 3554.4 ms. The terms ‘$\frac{\;Total\;image\;processing\;time\;}{Total\;number\;of\;processed\;images}$’ and model loading time in Equation (6) were constant for the same model, whereas the term ‘$\frac{Model\;loading\;time\;}{Total\;number\;of\;processed\;images}$’ was smaller with more images generated by the data augmentation, resulting in a faster processing speed with data augmentation. The model loading time should be part of model processing and cannot be excluded when processing speed evaluation.

(6) $$\begin{array}{cc} Processing\;speed= & \frac{Model\;loading\;time+Total\;image\;processing\;time}{Total\;number\;of\;processed\;images}\\ = & \frac{Model\;loading\;time\;}{Total\;number\;of\;processed\;images}+\frac{\;Total\;image\;processing\;time\;}{Total\;number\;of\;processed\;images}. \end{array}$$

A best model classification accuracy of 98.7% was achieved in this study (Table 3 and Table 5) and was comparable to that of other deep learning studies for cattle muzzle recognition, in which the accuracy was 98.9% [27,28] and 99.1% [29]. Despite the discrepancies of cattle breeds, rearing environments, data acquisition conditions, and network architecture, all these studies achieved the desired accuracy (>90%), which again proves the empowering object recognition ability of deep learning and suggests a suitable application of the deep learning technique for individual cattle identification.

The processing speed ranged from 28.3 to 678.2 ms/image (Table 3 and Table 5) and was also comparable to or faster than some previous studies: 32.0–738.0 ms/image [15], 77.5–1361.9 ms/image [23], and 368.3–1193.3 ms/image [30]. Interestingly, these studies used machine learning or digital image processing algorithms as indicated in Table 1, and these models were supposed to be relatively lightweight compared to the deep learning models, with a faster processing speed, but our study suggested the opposite. In addition to programming language and platform, computing hardware explained the processing speed performance, particularly for configurations that were less advanced than those listed in Section 2.3.

### 3.4. Identification Accuracy of Each Cattle

**Figure 5:**
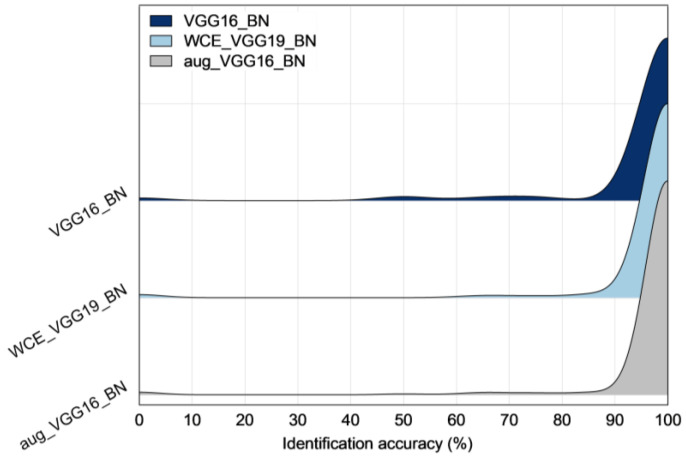
A ridgeline chart illustrating the probabilities of identification accuracy for individual cattle using (**top**) VGG16_BN without class imbalance optimization, (**middle**) VGG19_BN with weighted cross-entropy loss function, and (**bottom**) VGG16_BN with data augmentation.

A probability chart demonstrating the identification accuracy of individual cattle is presented in Figure 5 and summarized in Table 6. In general, 92.5–95.1% cattle were 100% accurately identified, suggesting a great potential of using deep learning techniques to identify individual cattle. The results are also in agreement with Table 5. Despite different models, the development with weighted cross-entropy and data augmentation indeed outperformed that with cross-entropy loss function only. Worst-case scenarios (with 0% identification accuracy) developed from models without class imbalance optimization were reduced from four to three with data augmentation. Best-case scenarios (100% identified) increased by 6–7 after class imbalance optimization. Accuracy, excluding best-case or worst-case scenarios, was improved by 1.3–1.5% after class imbalance optimization.

The ID numbers of cattle with 0% identification accuracy were 2100, 4549, 5355, and 5925, with only four cropped images per head (Table A1). Although more images per cattle may result in higher accuracy for identifying individual cattle [22], multiple factors should be considered for data collection, such as access to animals, resource availability, and labeling workload. An optimal threshold of images per head is favorable in balancing identification accuracy and difficulties associated with data collection. The commonly used per head image rate is 5–20, as suggested in Table 1. Our results also indicated that an animal with over four muzzle images for model development could be identified successfully (with over 90% accuracy) by deep learning models. These data suggest that five images per animal could be an appropriate threshold.

This project aims to initiate the very first step of an individual cattle identification system. Coupled with computer engineer and software development capacity, the optimal model, VGG16_BN, can be installed into a computer vision system to livestream cattle muzzles. In the future, such computer vision systems have the potential to be integrated into commercial beef cattle feedlot facilities via other facilities or technologies (e.g., hydraulic chute, mobile robot systems, drones, smartphones) that allow for individual cattle muzzle capture and maintain the consistency of data collection.

## 4. Conclusions

Individual beef cattle were identified with muzzle images and deep learning techniques. A dataset containing 268 US feedlot cattle and 4923 muzzle images was published along with this article, forming the largest dataset for beef cattle to date. A total of 59 deep learning models were comparatively evaluated for identifying muzzle patterns of individual cattle. The best identification accuracy was 98.7%, and the fastest processing speed was 28.3 ms/image. The VGG models performed better in terms of accuracy and processing speed. Weighted cross-entropy loss function and data augmentation could improve the identification accuracy for the cattle with fewer muzzle images. This study demonstrates the great potential of using deep learning techniques to identify individual cattle using muzzle images and to support precision beef cattle management.

## Figures and Tables

**Table 2:** Summary of deep learning image classification models evaluated in this study.

Model Name and Reference	Model Version	Highlighted Features	Total Parameters (Million)	Model Size (MB)
AlexNet [44]	AlexNet	First parallelization and distributed training with multiple GPUs.	61.1	221.6
DenseNet [45]	DenseNet121	Connections between each layer and every other layer in a feed-forward fashion. Numbers indicate that the model contains 121, 161, 169, and 201 layers in the networks.	8.1	28.2
	DenseNet161		29.0	104.4
	DenseNet169		14.3	50.3
	DenseNet201		20.2	72.3
DPN [46]	DPN68	Dual-path architecture; feature re-usage; new feature exploration; contains 68 layers.	13.0	46.2
EfficientNet [47]	EfficientNet_b0	Model scaling and balancing network depth, width, and resolution; neural architecture search; b0 to b7 correspond to input sizes of (256, 224), (256, 240), (288, 288), (320, 300), (384, 380), (489, 456), (561, 528), and (633, 600) pixels, respectively.	5.3	16.9
	EfficientNet_b1		7.8	26.6
	EfficientNet_b2		9.1	31.3
	EfficientNet_b3		12.2	42.9
	EfficientNet_b4		19.3	69.5
	EfficientNet_b5		30.4	111.2
	EfficientNet_b6		43.0	159.0
	EfficientNet_b7		66.3	247.6
Inception [48,49,50,51]	GoogleNet	Increasing the depth and width of the network while keeping the computational budget constant.	13.0	22.6
	InceptionV3	Factorized convolutions and aggressive regularization.	27.2	96.1
	InceptionV4	Combination of Inception architectures with residual connections.	42.7	159.1
	InceptionResNetV2		55.8	209.5
	Xception	Depth-wise separable convolutions; dubbed Xception.	22.9	81.8
MnasNet [52]	MnasNet_0.5	Automated mobile neural architecture search approach, model latency, mobile phones, and factorized hierarchical search space; 0.5 and 1.0 indicate the network with depth multipliers of 0.5 and 1.0.	2.2	5.1
	MnasNet_1.0		4.4	13.4
MobileNet [53,54]	MobileNetV2	Inverted residual structure, lightweight depth-wise convolutions, and maintaining representational power.	3.5	10.0
	MobileNetV3_Large	Hardware-aware network architecture search complemented by the NetAdapt algorithm. MobileNetV3-Large and MobileNetV3-Small target high- and low-resource use cases.	2.5	17.6
	MobileNetV3_Small		5.5	7.0
RegNet [55]	RegNetY_400MF	Parametrizing populations of networks, elevated design space level, quantized linear function, and a wide range of flop regimes; RegNetX indicates the network with the X block (a standard residual bottleneck block), and RegNetY indicates the network with the X block and Squeeze-and-Excitation networks; 400MF, 800MF, 1.6GF, 3.2GF, 8.0GF, 16GF, and 32GF represent networks with flop regimes of 400 MB, 800 MB, 1.6 GB, 3.2 GB, 8.0 GB, 16 GB, and 32 GB, respectively.	4.3	15.6
	RegNetY_800MF		6.4	22.6
	RegNetY_1.6GF		11.2	40.7
	RegNetY_3.2GF		19.4	70.4
	RegNetY_8.0GF		39.4	145.1
	RegNetY_16GF		83.6	311.1
	RegNetY_32GF		145.0	543.7
	RegNetX_400MF		5.5	20.2
	RegNetX_800MF		7.3	26.1
	RegNetX_1.6GF		9.2	32.8
	RegNetX_3.2GF		15.3	56.0
	RegNetX_8.0GF		39.6	146.1
	RegNetX_16GF		54.3	201.9
	RegNetX_32GF		107.8	405
ResNet [56]	ResNet18	Residual learning framework, shortcut connections, avoiding feature vanishing, and achieving decent accuracy in deeper neural networks; 18, 34, 50, 101, and 152 indicate networks with 18, 34, 50, 101, and 152 layers, respectively.	11.7	43.2
	ResNet34		21.8	81.9
	ResNet50		25.6	92.1
	ResNet101		44.5	164.8
	ResNet152		60.2	224.8
ResNeXt [57]	ResNeXt50_32×4d	Highly modularized network architecture, aggregating a set of transformations with the same topology, and cardinality; 50 and 101 refer to networks with 50 and 101 layers, respectively; 32 refers to networks with 32 paths/cardinalities in the widthwise direction; 4d and 8d refer to networks with 4 and 8 stages/depths of residual blocks.	25.0	90.1
	ResNeXt101_32×8d		88.8	334
ShuffleNet [58]	ShuffleNetV2_×0.5	Direct metric of computation complexity on the target platform, FLOPs; ×0.5 and ×1.0 refer to networks with 0.5× and 1.0× output channels, respectively.	1.4	2.5
	ShuffleNetV2_×1.0		2.3	6.0
SqueezeNet [59]	SqueezeNet_1.0	50× fewer parameters, and <0.5 MB model sizes; SqueezeNet_1.0 is the original network, while SqueezeNet_1.1 has 2.4× less computation and slightly fewer parameters than the original version.	1.2	3.4
	SqueezeNet_1.1		1.2	3.3
VGG [60]	VGG11	Increasing depth using an architecture with very small (3 × 3) convolution filters; 11, 13, 16, and 19 indicate networks with 11, 13, 16, and 19 layers, respectively; BN represents networks with batch normalization.	132.9	495.4
	VGG11_BN		132.9	495.5
	VGG13		133.0	496.1
	VGG13_BN		133.0	496.2
	VGG16		138.4	516.4
	VGG16_BN		138.4	516.5
	VGG19		143.7	536.6
	VGG19_BN		143.7	536.7
Wide ResNet [61]	Wide_ResNet50_2	Decreasing depth and increasing width of residual networks, and bottleneck network; 50 and 101 refer to networks with 50 and 101 layers, respectively; 2 is used to differentiate the network from ResNet.	68.9	257.4
	Wide_ResNet101_2		126.9	479.1

Note: GPU, graphical processing unit; DenseNet, densely connected network; DPN, dual-path network; EfficientNet, efficient network; MnasNet, mobile neural architecture search network; MobileNet, mobile network; RegNet, regular network; ResNet, residual network; ResNeXt, combination of residual network and next dimension; ShuffleNet, a highly efficient architecture with a novel channel shuffle operation; SqueezeNet, squeezed network; VGG very deep convolutional network developed by the Visual Geometry Group.

**Table 3:** Model performance parameters (testing accuracy, processing speed, and comprehensive index (Equation (3))) of individual beef cattle classification. The outperformed models for each parameter were highlighted in bold fonts.

Model	Accuracy (%)	Processing Speed (ms/Image)	CI	Model	Accuracy (%)	Processing Speed (ms/Image)	CI
AlexNet	96.5	36.0	7.8	RegNetY_32GF	94.7	564.0	22.6
DenseNet121	93.0	153.5	25.6	RegNetX_400MF	86.6	53.1	32.6
DenseNet161	94.7	278.6	21.6	RegNetX_800MF	84.6	70.0	36.2
DenseNet169	94.7	183.8	19.4	RegNetX_1.6GF	84.8	99.5	36.4
DenseNet201	94.6	224.4	21.6	RegNetX_3.2GF	86.6	142.0	35.2
DPN68	94.4	153.1	19.8	RegNetX_8.0GF	88.0	208.6	37.0
EfficientNet_b0	49.4	122.4	48.2	RegNetX_16GF	89.8	360.4	37.4
EfficientNet_b1	55.1	159.3	45.8	RegNetX_32GF	92.3	574.3	32.4
EfficientNet_b2	54.7	171.3	46.8	ResNet18	90.5	60.3	27.6
EfficientNet_b3	60.0	221.3	44.6	ResNet34	93.7	86.2	19.4
EfficientNet_b4	51.2	283.1	52.2	ResNet50	91.3	153.0	29.2
EfficientNet_b5	51.0	425.6	54.2	ResNet101	94.2	228.7	23.4
EfficientNet_b6	47.3	468.2	56.0	ResNet152	93.7	319.1	26.8
EfficientNet_b7	54.1	678.2	53.4	ResNeXt50_32×4d	93.0	180.4	25.6
GoogleNet	59.4	78.3	40.8	ResNeXt101_32×8d	96.1	419.6	18.8
InceptionV3	81.7	112.9	38.4	ShuffleNetV2_×0.5	1.2	32.3	47.4
InceptionV4	80.6	187.0	42.0	ShuffleNetV2_×1.0	1.3	43.3	47.2
InceptionResNetV2	66.9	244.7	44.8	SqueezeNet_1.0	95.0	62.1	12.6
Xception	58.3	207.0	45.6	SqueezeNet_1.1	95.9	45.3	9.8
MnasNet_0.5	2.9	46.2	46.8	VGG11	96.7	127.0	10.8
MnasNet_1.0	57.6	66.1	41.6	VGG11_BN	98.1	141.0	6.2
MobileNetV2	91.3	77.4	26.2	VGG13	98.0	175.9	9.4
MobileNetV3_Large	95.9	60.2	11.4	VGG13_BN	97.7	196.0	11.0
MobileNetV3_Small	93.2	35.6	18.8	VGG16	97.7	211.0	12.4
RegNetY_400MF	90.7	59.6	26.4	VGG16_BN	98.4	239.1	9.2
RegNetY_800MF	86.5	75.2	34.8	VGG19	97.1	248.0	14.6
RegNetY_1.6GF	88.8	103.8	32.6	VGG19_BN	98.1	276.6	11.8
RegNetY_3.2GF	91.6	150.5	27.4	Wide_ResNet50_2	89.6	243.7	36.6
RegNetY_8.0GF	92.1	269.8	30.8	Wide_ResNet101_2	90.4	404.4	37.0
RegNetY_16GF	93.6	370.3	28.0				

Note: CI, comprehensive index. Descriptions of the models are provided in Table 2.

**Table 4:** Pearson correlation coefficient (*R*) matrix among model total parameter and size, accuracy, and processing speed.

	Accuracy	Processing Speed
Total parameter	0.389	0.517
Model size	0.391	0.521

**Table 5:** Accuracy and processing speed for the 20 selected models before and after optimization for class imbalance. The outperformed models for each parameter were highlighted in bold fonts.

Model	Cross Entropy		Weighted cross Entropy		Data Augmentation		Model Loading Time (ms)
	Accuracy (%)	Processing Speed (ms/Image)	Accuracy (%)	Processing Speed (ms/Image)	Accuracy (%)	Processing Speed (ms/Image)	
AlexNet	96.5	36.0	95.8	36.3	95.7	29.7	95.7
DenseNet161	93.0	153.5	97.3	286.2	98.3	139.1	133.0
DenseNet169	94.7	278.6	97.6	176.1	97.9	90.2	807.2
DenseNet201	94.7	183.8	97.1	221.5	98.2	110.5	963.3
DPN68	94.6	224.4	97.8	151.8	98.6	80.5	1183.2
MobileNetV3_Large	94.4	153.1	97.4	61.6	95.2	39.8	261.2
MobileNetV3_Small	96.5	**36.0**	95.8	**35.9**	86.6	**28.3**	186.3
RegNetY_32GF	94.7	564.0	97.1	553.7	95.1	297.5	244.3
ResNet34	93.7	86.2	97.0	88.3	97.6	54.7	767.4
ResNeXt101_32×8d	96.1	419.6	98.0	419.1	98.5	210.7	2539.9
SqueezeNet_1.0	95.0	62.1	92.6	62.0	78.3	39.6	120.0
SqueezeNet_1.1	95.9	45.3	94.1	44.7	93.9	32.4	127.7
VGG11	96.7	127.0	96.5	128.2	97.2	77.8	3391.3
VGG11_BN	98.1	141.0	98.2	141.8	98.0	83.3	3237.8
VGG13	98.0	175.9	95.6	176.2	98.2	99.3	3227.4
VGG13_BN	97.7	196.0	97.9	199.6	98.5	109.5	3279.0
VGG16	97.7	211.0	96.9	213.4	97.4	117.9	3435.4
VGG16_BN	98.4	239.1	97.7	238.5	98.7	125.2	3414.0
VGG19	97.1	248.0	95.7	249.5	97.8	137.3	3525.2
VGG19_BN	98.1	276.6	98.5	274.8	97.8	159.6	3554.4
Average ± SD	96.1 ± 1.6	192.9 ± 129.8	96.7 ± 1.5	188.0 ± 131.5	95.9 ± 5.0	103.2 ± 66.5	1724.7 ± 1494.2

Note: ‘Cross-entropy’ indicates models developed with the cross-entropy loss function and without any class imbalance optimization. Descriptions of the models are provided in Table 2.

**Table 6:** Accuracy and processing speed before and after optimization for class imbalance.

Development Strategy	Number of Cattle with 0% Identification Accuracy	Number of Cattle 100% Accurately Identified	Accuracy (%, Excluding 100% and 0%)
Cross-entropy	4	248	96.2 ± 15.1
Weighted cross-entropy	4	254	97.5 ± 13.3
Data augmentation	3	255	97.7 ± 12.3

Note: The model used was VGG16_BN for cross-entropy and data augmentation and VGG19_BN for weighted cross-entropy.

## Data Availability

The data presented in this study are publicly available in Zenodo.org at http://doi.10.5281/zenodo.6324360.

## Appendix A

**Table A1:** Image counts and assigned weight in weighted cross-entropy loss function for each cattle.

Cattle ID	Image Counts	Weight	Cattle ID	Image Counts	Weight	Cattle ID	Image Counts	Weight	Cattle ID	Image Counts	Weight
0100	8	8.75	3812	12	5.83	4680	19	3.68	5143	9	7.78
0200	10	7.00	3814	13	5.38	4685	11	6.36	5153	5	14.00
0300	17	4.12	3819	19	3.68	4686	31	2.26	5164	32	2.19
0400	7	10.00	3832	42	1.67	4716	16	4.38	5165	10	7.00
0500	14	5.00	3842	14	5.00	4717	5	14.00	5170	40	1.75
0600	19	3.68	3844	15	4.67	4733	29	2.41	5171	20	3.50
0700	16	4.38	3847	21	3.33	4739	26	2.69	5197	14	5.00
0800	18	3.89	3852	29	2.41	4748	15	4.67	5207	18	3.89
0900	12	5.83	3856	16	4.38	4770	11	6.36	5208	4	17.50
1000	12	5.83	4208	18	3.89	4775	15	4.67	5215	48	1.46
1100	11	6.36	4259	6	11.67	4776	25	2.80	5224	28	2.50
1200	11	6.36	4323	19	3.68	4804	15	4.67	5234	8	8.75
1300	12	5.83	4326	10	7.00	4819	18	3.89	5235	24	2.92
1400	13	5.38	4330	28	2.50	4820	38	1.84	5249	24	2.92
1500	6	11.67	4339	20	3.50	4833	26	2.69	5273	18	3.89
1600	14	5.00	4347	19	3.68	4839	26	2.69	5275	14	5.00
1700	12	5.83	4363	21	3.33	4840	16	4.38	5282	6	11.67
1800	22	3.18	4369	16	4.38	4895	24	2.92	5283	14	5.00
1900	8	8.75	4381	24	2.92	4915	30	2.33	5297	32	2.19
2000	14	5.00	4385	23	3.04	4921	14	5.00	5298	25	2.80
2100	4	17.50	4399	7	10.00	4947	15	4.67	5307	10	7.00
2200	6	11.67	4421	32	2.19	4951	39	1.79	5314	13	5.38
2220	6	11.67	4422	22	3.18	4969	12	5.83	5325	36	1.94
2300	22	3.18	4451	7	10.00	4971	11	6.36	5355	4	17.50
2320	14	5.00	4454	26	2.69	4984	24	2.92	5359	10	7.00
2400	23	3.04	4456	29	2.41	4985	11	6.36	5360	35	2.00
2500	33	2.12	4479	25	2.80	4986	26	2.69	5362	18	3.89
2510	10	7.00	4488	11	6.36	4995	6	11.67	5373	27	2.59
2600	27	2.59	4499	29	2.41	5009	17	4.12	5374	27	2.59
2700	17	4.12	4537	12	5.83	5026	23	3.04	5403	26	2.69
2710	15	4.67	4539	18	3.89	5028	21	3.33	5404	22	3.18
2740	8	8.75	4545	29	2.41	5066	14	5.00	5407	40	1.75
2800	24	2.92	4549	4	17.50	5073	14	5.00	5408	18	3.89
2900	15	4.67	4551	28	2.50	5077	16	4.38	5410	20	3.50
2930	6	11.67	4568	23	3.04	5083	29	2.41	5411	31	2.26
3000	15	4.67	4607	34	2.06	5090	18	3.89	5425	26	2.69
3100	13	5.38	4613	70	1.00	5097	25	2.80	5427	13	5.38
3200	16	4.38	4614	25	2.80	5100	8	8.75	5432	19	3.68
3300	13	5.38	4649	34	2.06	5112	14	5.00	5477	9	7.78
3400	7	10.00	4668	21	3.33	5132	30	2.33	5507	25	2.80
3420	4	17.50	4678	19	3.68	5133	12	5.83	5508	25	2.80
3802	8	8.75	4679	16	4.38	5138	11	6.36	5509	22	3.18
5519	25	2.80	5781	27	2.59	6124	10	7.00	6295	10	7.00
5529	29	2.41	5784	16	4.38	6161	18	3.89	6313	34	2.06
5537	37	1.89	5803	15	4.67	6167	21	3.33	6331	32	2.19
5556	8	8.75	5804	26	2.69	6171	12	5.83	6333	54	1.30
5559	21	3.33	5806	14	5.00	6184	16	4.38	6442	13	5.38
5581	29	2.41	5809	24	2.92	6189	12	5.83	6446	8	8.75
5604	14	5.00	5815	14	5.00	6191	18	3.89	6458	10	7.00
5605	14	5.00	5816	9	7.78	6196	17	4.12	6479	15	4.67
5620	12	5.83	5836	22	3.18	6197	12	5.83	6499	9	7.78
5630	4	17.50	5844	30	2.33	6199	14	5.00	6505	14	5.00
5633	13	5.38	5886	25	2.80	6210	10	7.00	6506	18	3.89
5634	13	5.38	5925	4	17.50	6213	12	5.83	6530	10	7.00
5639	12	5.83	5932	38	1.84	6216	18	3.89	6606	12	5.83
5654	53	1.32	5953	30	2.33	6220	12	5.83	8050	4	17.50
5658	12	5.83	5971	45	1.56	6226	15	4.67	8094	10	7.00
5670	16	4.38	5986	5	14.00	6237	6	11.67	8095	8	8.75
5677	12	5.83	6011	32	2.19	6253	8	8.75	9021	10	7.00
5695	20	3.50	6012	19	3.68	6266	10	7.00	9029	29	2.41
5697	31	2.26	6017	12	5.83	6276	15	4.67	9634	31	2.26
5717	16	4.38	6022	8	8.75	6277	12	5.83	9635	8	8.75
5745	15	4.67	6038	22	3.18	6278	13	5.38	9736	18	3.89
5761	10	7.00	6066	50	1.40	6282	12	5.83	9742	19	3.68
5762	7	10.00	6071	26	2.69	6283	5	14.00	9773	10	7.00
5774	16	4.38	6084	36	1.94	6287	14	5.00	9798	42	1.67
5777	12	5.83	6098	20	3.50	6294	10	7.00	9801	10	7.00
